# Serum Metabolite Profiles in Participants of Lung Cancer Screening Study; Comparison of Two Independent Cohorts

**DOI:** 10.3390/cancers13112714

**Published:** 2021-05-31

**Authors:** Piotr Widłak, Karol Jelonek, Agata Kurczyk, Joanna Żyła, Magdalena Sitkiewicz, Edoardo Bottoni, Giulia Veronesi, Joanna Polańska, Witold Rzyman

**Affiliations:** 1 Maria Sklodowska-Curie National Research Institute of Oncology, Gliwice Branch, 44-102 Gliwice, Poland; karol.jelonek@io.gliwice.pl (K.J.); agata.kurczyk@io.gliwice.pl (A.K.); 2 Silesian University of Technology, 44-100 Gliwice, Poland; joanna.zyla@polsl.pl (J.Ż.); joanna.polanska@polsl.pl (J.P.); 3 Medical University of Gdansk, 80-210 Gdansk, Poland; magdalena.sitkiewicz@gumed.edu.pl (M.S.); wrzyman@gumed.edu.pl (W.R.); 4 Humanitas Research Hospital, Rozzano, 20089 Milan, Italy; emanuele.bottoni@humanitas.it; 5 Vita-Salute San Raffaele University, 20132 Milan, Italy; veronesi.giulia@hsr.it; 6 IRCCS San Raffaele Scientific Institute, 20132 Milan, Italy

**Keywords:** biomarkers, early detection, metabolomics, lung cancer, screening study

## Abstract

**Simple Summary:**

Serum metabolite profiles were compared in healthy participants and lung cancer individuals in two independent screening studies. A reduced level of lipids, particularly cholesteryl esters, was observed in cancer patients. Despite several compounds showing significant differences between cancer patients and healthy controls within each study, only a few cancer-related features were common when both studies were compared, including reduced levels of LPC(18:0). A large heterogeneity of serum metabolomes was observed, both within and between studies, which impaired the accuracy of classifiers based on specific metabolites.

**Abstract:**

Serum metabolome is a promising source of molecular biomarkers that could support early detection of lung cancer in screening programs based on low-dose computed tomography. Several panels of metabolites that differentiate lung cancer patients and healthy individuals were reported, yet none of them were validated in the population at high-risk of developing cancer. Here we analyzed serum metabolome profiles in participants of two lung cancer screening studies: MOLTEST-BIS (Poland, *n* = 369) and SMAC-1 (Italy, *n* = 93). Three groups of screening participants were included: lung cancer patients, individuals with benign pulmonary nodules, and those without any lung alterations. Concentrations of about 400 metabolites (lipids, amino acids, and biogenic amines) were measured by a mass spectrometry-based approach. We observed a reduced level of lipids, in particular cholesteryl esters, in sera of cancer patients from both studies. Despite several specific compounds showing significant differences between cancer patients and healthy controls within each study, only a few cancer-related features were common when both cohorts were compared, which included a reduced concentration of lysophosphatidylcholine LPC (18:0). Moreover, serum metabolome profiles in both noncancer groups were similar, and differences between cancer patients and both groups of healthy participants were comparable. Large heterogeneity in levels of specific metabolites was observed, both within and between cohorts, which markedly impaired the accuracy of classification models: The overall AUC values of three-state classifiers were 0.60 and 0.51 for the test (MOLTEST) and validation (SMAC) cohorts, respectively. Therefore, a hypothetical metabolite-based biomarker for early detection of lung cancer would require adjustment to lifestyle-related confounding factors that putatively affect the composition of serum metabolome.

## 1. Introduction

Lung cancer is an aggressive, usually asymptomatic disease with a high mortality rate that is the leading cause of cancer-related deaths. Every year, nearly two million people die from this cancer worldwide. Late diagnosis and its aggressive character determine the poor outcome of this malignancy. In about 70% of patients, lung cancer is detected in advanced stages, which precludes radical local treatment and worsens prognosis (the average 5-year survival of about 10–20%). However, in the case of the disease detected at the early stages, the prognosis is much better (the average 5-year survival varies between 65 and 85%) [1]. Primary prevention (the reduction of consumption of tobacco products) is of utmost importance, but early detection (secondary prevention) is currently the only way to defeat this disease. Early lung cancer detection became a reality in this decade due to the introduction of low-dose computed tomography (LDCT) lung cancer screening [2]. This test significantly reduces lung cancer mortality, which was demonstrated for the first time by the NLST study conducted in the USA (years 2001–2010), showing a 20% reduction in lung cancer mortality in the group subjected to the LDCT [3]. The potential of LDCT screening programs to reduce lung cancer mortality was further confirmed by the European NELSON study [4] and the Danish Lung Cancer Screening Trial [5]. However, currently conducted LDCT tests detect in most screens unidentified subcentimeter nodules (the so-called indeterminate pulmonary nodules, IPN), which lead to a significant percentage of false-positive results (positive predictive value of 3.8% in the NLST). Therefore, a large fraction of individuals with lung nodules detected by the LDCT test undergo futile diagnostic and therapeutic procedures [6]. This increases the socioeconomic implication in the form of higher costs and possible harm for screened individuals. Hence, higher specificity of the screening test should result in higher diagnostic accuracy and cost-effectiveness, which would be possible by supplementing the LDCT with other auxiliary diagnostic tests that could either preselect individuals for LDCT examination or discriminate between benign and malignant chest abnormalities detected by LDCT [7,8].

A promising candidate for the support of LDCT in lung cancer screening is a biomarker-based molecular test. The hypothetical biomarker of early lung cancer could be detected in the blood, which is a particularly valuable source of molecular information on disease-related processes, with many actual and prospective applications as a “liquid biopsy” of cancer. For over a decade, intensive studies to identify new biomarkers of early lung cancer were focused on various components of the blood, including circulating tumor cells (CTC), circulating free DNA (cfDNA), autoantibodies, and components of the proteome, peptidome, and transcriptome of serum/plasma [9,10,11,12]. Moreover, cancer-related metabolites present in the blood appeared as an emerging source of biomarkers for the detection and diagnosis of different malignancies [13], including lung cancer. Several studies reported signatures of serum/plasma metabolites that differentiated patients with lung cancer from healthy individuals or patients with nonmalignant lung diseases. Examples of serum metabolites with a potential discriminating value are choline-containing phospholipids and sphingolipids [14,15,16]. Two relatively large studies using NMR-based analysis of plasma or serum metabolome revealed a promising diagnostic potential of multicomponent lung cancer signatures built of different types of small metabolites [17,18]. Another study, using MS-based approaches, revealed a large set of metabolites whose serum levels discriminated lung cancer patients from matched controls and allowed for building multicomponent cancer classifiers [19]. However, lung cancer patients enrolled in the above-mentioned studies included both early and advanced clinical cancer cases, thus the potential relevance of proposed signatures for early detection of lung cancer and their potential applicability in lung cancer screening remains to be verified.

Recently, our group published the results of a pilot study aimed to identify serum metabolites that differentiated patients with screening-detected lung cancer from healthy participants of the Gdansk Lung Cancer Screening Study [20,21]. Here, we aimed to extend and validate these findings using a larger set of samples collected in the frame of two single-center screening studies: MOLTEST in Gdansk, Poland (*n* = 369) and SMAC in Milano, Italy (*n* = 93). Three balanced groups of individuals (lung cancer cases, benign lung nodules, and healthy controls) were included and their serum metabolite profiles were compared using a comprehensive quantitative MS-based approach.

## 2. Materials and Methods

### 2.1. Study Subject

A major part of the material included in this study (test cohort) was collected during the MOLTEST-BIS Lung Cancer Screening Program performed by the Medical University of Gdansk between 2015 and 2018. This program enrolled over 6000 participants and offered LDCT examinations for current or former smokers with at least a 20 pack-year history, aged from 50 to 75 years. This report involves three groups of participants of the MOLTEST study (123 individuals in each group): (i) patients who were ultimately diagnosed with lung cancer, (ii) participants with CT-detected lung nodules that were confirmed benign by histopathology, and (iii) participants with no CT-detected lung nodules that have no other cancer-related health problem. Groups were matched according to age and smoking history. Furthermore, part of the material included in this study (validation cohort) was collected during the Smokers Health Multiple Action (SMAC) study performed by the Humanitas Clinical and Research Center, Milano between 2018 and 2021. This study included about 2000 participants (inclusion criteria: age = 55 years old and exposure to smoking more than 30 packs-year). This report involves three groups of participants of the SMAC-1 study (31 individuals in each group). The characteristics of all groups are presented in Table 1; all cancer cases available in both studies were included together with matched controls from both noncancer groups. Studies were approved by the appropriate Ethics Committees (Medical University of Gdansk, approval no. NKBBN/376/2014, and the Humanitas Clinical and Research Center, approval no. CE Humanitas ex D.M. 390/18), and all participants provided informed consent indicating their voluntary participation in the project and provision of blood samples for future research.

### 2.2. Serum Sample Preparation

Peripheral blood was collected into a 5 mL BD Vacutainer Tube, incubated for 30 min at room temperature to allow clotting, and then centrifuged at 1000× *g* for 10 min to remove the clot. The serum was aliquoted and stored at −80 °C before further processing.

### 2.3. Targeted Metabolomics

Ten µL of serum was analyzed by a targeted quantitative approach using a combined direct flow injection and liquid chromatography (LC) high-resolution mass spectrometry (HRMS) assay using the Absolute IDQ p400 HR kit (test plates in the 96-well format; Biocrates Life Sciences AG, Innsbruck, Austria) according to the manufacturer’s protocol. This strategy hypothetically allows simultaneous quantification of 407 metabolites or their isomer groups: 42 amino acids and biogenic amines, 55 acylcarnitines, 60 di- and triglycerides, 196 (lyso)phosphatidylcholines, 40 sphingolipids, 14 cholesteryl esters, and hexose. The method combines derivatization and extraction of analytes with selective mass-spectrometric detection using integrated isotope-labeled internal standards absolute quantification. Mass spectrometry analyses were carried out on Orbitrap Q Exactive Plus (Thermo Fisher Scientific, Waltham, MA, USA) equipped with a 1290 Infinity UHPLC (Agilent, Santa Clara, CA, USA) system using an Agilent Zorbax Eclipse XDB-C18 (3.5 μm) 3.0 × 100 mm column and controlled by Xcalibur 4.1. software. The acquired data were processed using Xcalibur 4.1. and MetIDQ DB110-2976 (Biocrates Life Sciences AG) software. Concentrations of all metabolites were calculated in μM.

### 2.4. Data Normalization

Two types of missing values in the metabolomics dataset were detected: measurements lacking due to the internal calibrant error and “zero” values below the level of detection/quantitation. Ten and fifty percent missing values in each patient’s group were allowed for either type of error [22], respectively; otherwise, the compound was excluded from further analyses. Measurements missing due to the calibrant error were filled by values imputed using the k-nearest neighbor approach [23]); the nearest observed data were identified using correlation distance metric, and the mean value of the three nearest neighbors was used (based on measurements collected for the same patient’s group using the same test plate). “Zero” values were replaced with random numbers generated from normal distribution truncated to a segment between 0 and the limit of detection/quantitation for a given test plate (according to the modified method described in [22]). The final dataset comprised 385 metabolites where missing values were imputed. After the imputation of the missing values, the dataset was batch corrected using an empirical Bayes method [24]; we assumed that samples measured using one multiple sample plate represent one batch. Before batch adjustment, data were transformed using the log base 2 function. Batch-corrected data were analyzed using the nonlinear dimension reduction algorithm (UMAP) for the dataset structure visualization [25].

### 2.5. Statistical Analyses

To estimate the significance of differences in levels of metabolites used in quantitative analyses (259 compounds with less than 50% initial “zero” values in each group), the Kruskal−Wallis test was applied, followed by the posthoc Conover test for pairwise comparisons [26]. Moreover, the eta-squared effect size was calculated for Kruskal−Wallis, whereas the Conover test statistic was standardized by the square root of the sample’s size which interpretation corresponds to Pallant “r” effect size [27]. Additionally, the Jonckheere−Terpstra test [28] was evaluated to investigate the trend across order groups. Finally, the Lancaster probability integration method [29] was used to combine results from the Kruskal−Wallis test between cohorts. Furthermore, the chi-square independence test was applied to test whether the absence/presence status of the remaining 126 compounds was a group-related feature. The Benjamini−Hochberg procedure for the FDR correction was applied when necessary. All statistical hypotheses were tested at the 5% significance level.

### 2.6. Sample Classification

The classification model was constructed using the multinomial logistic regression (MLR) approach. The 10-fold cross-validation (10-CV) was applied. At each fold, the test set was extracted from the MOLTEST cohort by taking ten samples from each group, then the internal multiple random cross-validation (MRCV) procedure was applied for the remaining samples. The MRCV steps were as follows: (i) data split intro train (70%) and test (30%) subsets, (ii) forward feature selection for MLR on train subset with stop criterion ΔBIC ≤ 2, (iii) classification of predicted probability to one of the possible groups by maximum a posteriori, (iv) evaluation of accuracy on the train and test subset. The MRCV procedure was repeated 100 times at each fold. The resulting set of 100 models served for feature ranking generation. Features (i.e., metabolites) included in each model were sorted by their order of addition in the forward procedure, then the elbow technique was used to extract the most relevant compound for the final MLR model. Finally, the classification parameters were evaluated using the initial MOLTEST test set and validated using the SMAC set. The overall accuracy, AUC, sensitivity, specificity, and balanced accuracy were calculated at each fold of 10-CV, and the mean values were calculated with a 95% confidence interval (CI).

## 3. Results

**Figure 1:**
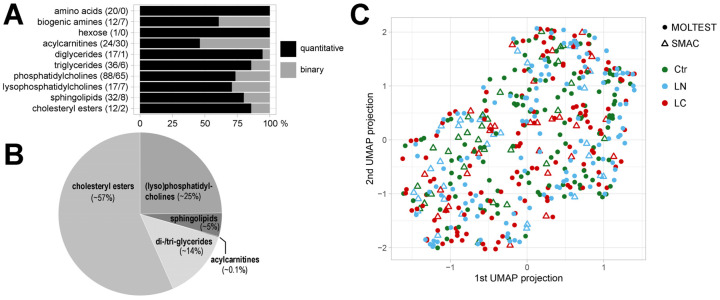
General characterization of the serum metabolite profile: (**A**)—numbers of metabolites in different classes used in quantitative and binary analyses (chart shows the relative contribution of compounds used in either type of analysis). (**B**)—relative contribution of different classes of lipids to the aggregated concentration of whole detected lipids. (**C**)—the global structure of the dataset. Spatial visualization was created using the UMAP data transformation from 385-dimensional metabolic space to 2D view, preserving the structure of the high-dimensional data to explore the potential sample clustering. Samples from MOLTEST and SMAC cohorts are marked with circles and triangles, respectively (Ctr—controls, LN—benign lung nodules, LC—lung cancer).

Metabolite profiles were established in a set of 462 serum samples collected from participants of two independent lung cancer screening studies (Table 1). There were 385 metabolites detected by a mass spectrometry-based approach, among which 259 metabolites were quantified in the majority of samples and used in quantitative analyses, including 32 amino acids and biogenic amines, one sugar (hexose), and 226 lipids or their isotope groups (24 acylcarnitines, 53 glycerides, 105 glycerophospholipids, 32 sphingolipids, and 12 cholesteryl esters). This subset of metabolites consisted of 117 high-abundance compounds quantified in all samples and 142 compounds where data imputation methods were used to find missing values, which were used in further quantitative analyses (Figure 1A). We found that cholesteryl esters (i.e., major components of the so-called total cholesterol), whose amount represented more than half of detected lipids, were the most abundant group of serum metabolites. Other abundant groups of lipids were choline-containing glycerophospholipids and glycerides, which represented about 25% and 14% of total serum lipids, respectively (Figure 1B). A whole set of detected metabolites was analyzed by an unsupervised approach to detect a potential “global pattern” of differences among both included cohorts (MOLTEST and SMAC) and three compared groups of patients: participants with no LDCT-detected lung abnormalities (Ctr), participants with LDCT-detected lung nodules that were confirmed benign by histopathology (LN), and patients who were ultimately diagnosed with lung cancer (LC). The data were transformed by the UMAP dimension reduction tool from a 385-dimensional metabolic space to a 2-dimensional view with preservation of the dataset structure. Low-dimensional data projection allowed the exploration of the global spatial dataset structure and thus potential sample clustering. We found a large interindividual heterogeneity of samples and no separation of either the three patient groups or the two cohorts was observed, which is illustrated in Figure 1C.

**Figure 2:**
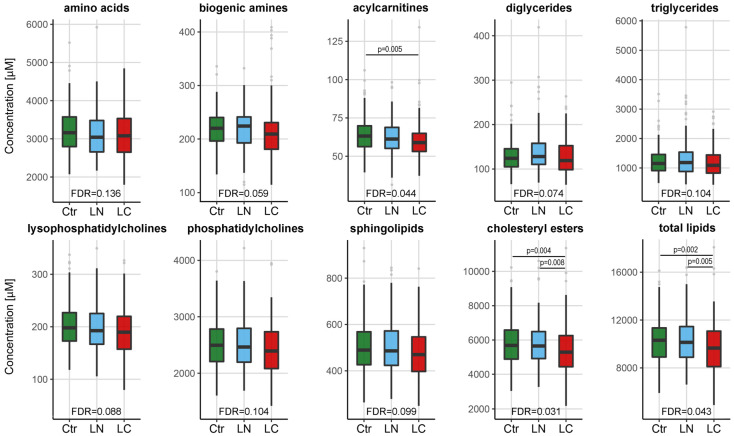
Aggregated concentrations of different classes of metabolites in serum samples of healthy controls (Ctr), individuals with benign lung nodules (LN), and lung cancer patients (LC). Boxplots show the minimum and maximum values, lower and upper quartile, and median (outliers are marked with gray circles); represented are FDR-corrected results of the Kruskal−Wallis test for a general variance and the posthoc test *p*-values for a significance of differences between pairwise compared groups (*n* = 154 in each group).

The set of 259 quantitated serum metabolites was used to detect potential differences between three groups of the screening participants in both cohorts (MOLTEST and SMAC) analyzed together (154 individuals in each group). To estimate the significance of differences among these groups, the Kruskal−Wallis analysis of variance test was applied, followed by posthoc pairwise comparisons (data presented in Table S1). The total (aggregated) serum concentration of 10 classes of metabolites is depicted in Figure 2. In general, we observed a reduced concentration of lipid class, but not amino acids or biogenic amines class, in serum samples of lung cancer patients. Further, the difference in the total concentration of cholesteryl esters was also statistically significant between cancer patients and the two other groups of individuals (i.e., healthy controls and individuals with benign lung nodules). Then, we searched for specific metabolites in which concentration was significantly different between groups. There were 23 metabolites (from all classes but amino acids) whose levels showed statistically significant differences between groups of patients (corrected *p*-value < 0.05). These included 22 metabolites that showed differences between cancer patients and healthy controls, 17 metabolites that showed differences between cancer patients and individuals with benign lung nodules, and 2 metabolites that showed differences between healthy controls and individuals with benign lung nodules (pairwise test *p*-value < 0.05; details in Table S1).

However, only a few of these compounds showed similar differences among groups when cohorts from two different screening programs were analyzed separately, which is discussed in more detail in the next paragraph. Additionally, the level of 126 low-abundance metabolites was below the detection threshold in more than 50% of samples. These compounds were used in the binary-type of analysis (i.e., present vs. absent) aimed to detect potentially different distributions of missed measurements. However, none of these metabolites revealed statistically significant differences between groups of patients (Table S2). 

**Figure 3:**
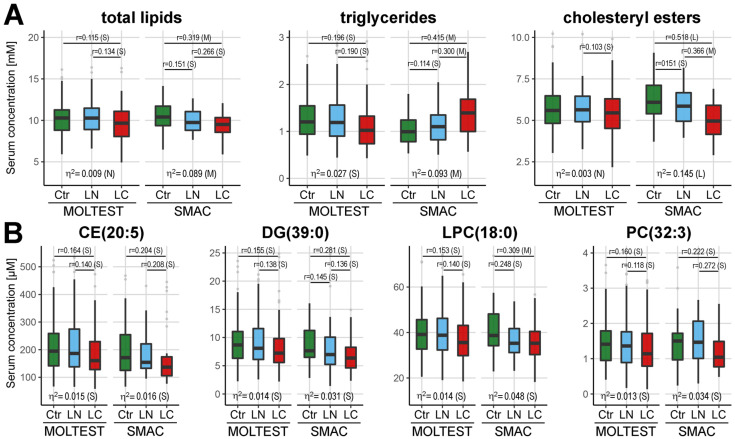
Levels of selected metabolites separately in two cohorts participating in lung cancer screening programs: (**A**)—aggregated concentration of total lipids, triglycerides, and cholesteryl esters in serum samples of healthy controls (Ctr), individuals with benign lung nodules (LN), and lung cancer patients (LC). (**B**)—concentration of selected compounds in serum samples from three analyzed groups. Boxplots show the minimum and maximum values, lower and upper quartile, and median (outliers are marked with gray circles); represented are the eta squared effect size values for a general variance and the Pallant r effect size for a significance of differences between pairwise compared groups (*n* = 123 and 31 in each group for MOLTEST and SMAC cohort, respectively); significance levels of effect size: N—negligible, S—small, M—medium, L—large.

The analysis of differences between the three groups was also performed separately for the MOLTEST and SMAC cohorts, then differentiating metabolites were revealed after the integration of both datasets. Considering a different number of samples in either cohort (123 and 31 samples per group, respectively) the effect size was used to estimate the significance of differences (Table S3). A reduced level of total serum lipids was observed in cancer samples from both subsets of individuals, yet different lipid classes seemed to contribute to this general observation (Figure 3A).

A significantly reduced level of cholesteryl esters was characteristic for cancer patients from the SMAC subset but not the MOLTEST subset, where this difference, though observed, was less significant (notably, controls have lower levels of cholesteryl esters in the Polish cohort than in the Italian cohort). On the other hand, a significantly reduced level of triglycerides was characteristic for cancer patients from the MOLTEST subset, while the overall level of TGs increased in samples of cancer patients from the SMAC subset. Important discrepancies between the MOLTEST and SMAC subsets were noted also for specific compounds. Nineteen out of twenty-three differentiating metabolites mentioned in the earlier paragraph remained significant only in one subset of patients or the trend for differences was opposite in both subsets. This could be exemplified by asparagine (Asn), which was significantly downregulated in MOLTEST cancer samples and upregulated in SMAC cancer samples. Only four metabolites showed coherence between both datasets: LPC(18:0), PC(32:3), DG(39:0), and CE(20:5). All these compounds were significantly downregulated (effect size medium or small) in samples of lung cancer patients from both MOLTEST and SMAC subsets (Figure 3B). Hence, a large part of the differences between cancer and control samples appeared to be specific for the patients’ cohort, yet a few of them seemed to be more universal. 

In the final step, the set of 259 quantitated metabolites was used to test and validate the multicomponent signature aimed to classify patients with lung cancer and benign lung nodules based on the levels of specific compounds. Samples collected in the MOLTEST study were used respectively as the training and test sets while samples collected in the SMAC study were used as the independent validation set. The three-class model (Ctr vs. LN vs. LC) was analyzed. The training step allowed the establishment of the rank of features (i.e., quantitated metabolites) that were the most important for classification, and the feature with maximum deviation in the elbow plot was the cut point at each cross-validation. Tested signatures included from 7 to 11 components; PC(41:5) and TG(52:4) were selected to the classification model in each iteration of 10-CV, while PC(38:6), PC(40:6), and SM(38:1) were selected in at least 5 out of 10 iterations. The overall mean accuracy of obtained models was 48% (compared to 33.3% expected by chance in a 3-class model) in the training set (with the mean AUC = 0.59). The indices of the classification models were further reduced in the test set (accuracy 37%; AUC = 0.60 with 95% CI: 0.56–65) and the validation set (accuracy 29%; AUC = 0.51 with 95% CI: 0.47–0.55), which indicated the insufficient power of the classification models. The corresponding levels of sensitivity and specificity are also presented in Table 2. The accuracy of classification in each group and the stability of classification results are presented Table S4. Hence, one should conclude that large interindividual variance within compared groups (as visible in Figure 1C) ultimately translated into a relatively low performance of the classification models built to predict a sample identity based on signatures composed of specific serum metabolites.

## 4. Discussion

Several studies already reported serum/plasma metabolome signatures that differentiated patients with lung cancer from healthy individuals or patients with nonmalignant lung abnormalities, including a few studies that analyzed relatively large groups and addressed wide panels of metabolites [17,18,19]. However, this current work is the first that involves a large group of individuals participating in early lung cancer screening studies and includes screen-detected cancer cases. Moreover, participants of two different lung cancer screening studies were compared, which revealed the potential influence of diet/lifestyle factors on the metabolome-based cancer signature (putative differences in genetic/ethnic background were less important between Polish and Italian cohorts). 

Our primary observation was a generally reduced level of lipids in sera of cancer patients when total levels of cholesteryl esters, glycerides, and choline-containing phospholipids were addressed (similarly, the level of acylcarnitines, a minor component of serum metabolome, was also reduced in cancer samples). The main fraction of lipids detected by our experimental system (approx. 60% of detected lipids) are cholesteryl esters that usually represent about 80% of total serum cholesterol (free unesterified cholesterol was not a target in our multiplex assay). The total amount of cholesteryl esters was readily reduced in cancer samples. It is noteworthy that the relationship between the profile of serum lipids, cholesterol in particular, and lung cancer risk has been known for decades. Several population studies documented that the risk of lung cancer is correlated with serum cholesterol levels (both total cholesterol and HDL cholesterol). This phenomenon was initially reported in the seven countries study [30], which showed that the risk of dying from lung cancer is higher for individuals with lower than average levels of serum cholesterol. This inverse correlation was confirmed in several other studies based on populations with different ethnic/genetic backgrounds [31], which suggested the general relevance of this factor. Moreover, the Lipid Research Clinics Coronary Primary Prevention Trial reported that serum cholesterol level decreased about 2 years before cancer diagnosis, which suggested the etiological role of cholesterol metabolism disturbances in lung cancer development [32]. Further, a few studies reported a positive correlation between the risk of lung cancer and serum/plasma level of triglycerides (TGs) [31], while other studies showed a U-shaped correlation [33] or even reduced level of TGs in the serum of lung cancer patients [34]. Hence, the association between lung cancer and glycerides’ level is more complex and could reflect interpopulation differences. We observed a reduced level of cholesteryl esters in the serum of lung cancer patients from both cohorts (yet the difference between healthy controls and cancer patients was higher in the Italian group), which was coherent with previous studies that generally showed an inverse correlation between serum cholesterol level and risk of lung cancer. The unforeseen situation was observed for the TGs level, which was reduced in cancer samples from the Polish group yet increased in cancer samples from the Italian group. This indicated that though disturbed metabolism of cholesterol that results in its reduced serum level was universally associated with lung cancer, the connection between the metabolism of glycerides and lung malignancy was putatively modulated by factors specific for different populations.

We noted that several putative cancer-related features were specific for either Polish and Italian cohorts, which indicated the influence of diet and other lifestyle factors on the serum metabolome (the influence of genetic and ethnic background was less likely). However, a few metabolites similarly discriminated against cancer and control samples in both cohorts, namely LPC(18:0), PC(32:3), DG(39:0), and CE(20:5). Among them, the lysophosphatidylcholine with stearic acid chain, i.e. LPC(18:0), has been already associated with cancer risk. A prospective study that addressed a correlation between serum metabolites and risk of breast, prostate, and colorectal cancers revealed that a higher serum level of LPC(18:0) was associated with a reduced risk of these cancers [35]. Moreover, LPC(18:0) was among the metabolites that were downregulated in lung cancer patients compared with healthy controls in studies based on small groups of clinical cancer cases [36,37]. Our pilot metabolomics study based on participants of the Gdańsk Lung Cancer Screening Study (2008–2010) also revealed a reduced level of LPC(18:0) in sera of cancer patients compared to healthy participants of the screening [21]. Reduced level of LPCs in the blood of cancer patients could reflect their transfer to tumor tissue and higher consumption by cancer cells, where they deliver respective fatty acids. Notably, an increased level of stearic acid was observed in lung tumors compared to normal lung tissues [38]. In general, the metabolism of phosphatidylcholines is significantly disturbed in cancer cells, hence the changed serum levels of their precursors (e.g., choline) and/or derivatives (e.g., lysophosphatidylcholines) are considered promising cancer markers [39]. Therefore, reduced serum level of LPC(18:0) appeared a universal feature of individuals with lung malignancy, with potential applicability for early lung cancer detection.

Generally, the same metabolites showed significantly different levels between cancer patients and participants of the screening study either without any lung abnormalities or with benign lung nodules. Moreover, only a few differences were noted between the latter two groups, independent of the screening cohort. Therefore, one could conclude that putative metabolic features associated with the presence of benign lung nodules do not result in specific changes that could be observed in the blood of smokers from the lung cancer high-risk group. On the other hand, metabolic features associated with the malignancy resulted in changes in the blood metabolome, which were not observed in the blood of high-risk smokers with benign lung nodules. Hence, a hypothetical metabolome-based biomarker could be used both for “preselection” of individuals at risk before LDCT examination and the “differentiation” of individuals with benign and malignant lung nodules. However, our study revealed large heterogeneity of serum metabolomes that resulted in low accuracy of classification models based on specific metabolites. This heterogeneity could result from various lifestyle-related factors, a phenomenon that was addressed in recent large metabolomics studies [40,41]. This aspect was not addressed specifically in the current study, yet putative lifestyle-related differences between populations (represented by Polish and Italian cohorts in our study) reduced the accuracy of serum metabolome-based signatures. Nevertheless, detailed information about nutritional habits and other lifestyle-related factors would be a valuable supplement to questionnaires for screening participants to facilitate the use of metabolome-based biomarkers in future studies. 

## 5. Conclusions

We observed a reduced total level of lipids, in particular cholesteryl esters, in sera of cancer patients from two independent screening studies. Moreover, differences between cancer patients and participants with no lung alterations or benign lung nodules were similar and only a few differences were noted between the latter two groups, indicating that putative metabolome-based signature could be used for both detection of cancer and diagnosis of indeterminate pulmonary nodules. However, despite several specific compounds showing significant differences between cancer patients and healthy controls within each study, only a few cancer-related features were common when both cohorts were compared. This included a reduced concentration of LPC(18:0), lysophosphatidylcholine, whose increased level was associated with a reduced risk of other solid cancers. A large variation in levels of specific metabolites was observed, both within and between cohorts, which markedly impaired the accuracy of classification models. Hence, the awaited “universal” metabolic biomarker of early lung cancer remains a challenge due to the large heterogeneity of serum metabolomes, putatively associated with lifestyle-related factors. Nevertheless, the signature of serum metabolites might be considered as a “local” solution if potential confounding factors were properly addressed.

## Figures and Tables

**Table 1:** Characteristics of donor cohorts.

Group	Healthy Controls	Benign Lung Nodules	Lung Cancer Cases
**Polish Cohort (MOLTEST)**			
*n* =	123	123	123
Clinical stage:	-	-	
- IA			49
- IB			10
- IIA			9
- IIB			10
- IIIA			17
- IIIB			7
- IVA			16
- IVB			5
Histopathology:	-	-	
- Adenocarcinoma			61
- Squamous cell carcinoma			35
- Not otherwise specified NSCLC			15
- SCLC			6
- Other cancer (incl. mixed types)			6
Sex:			
- Female	53	57	56
- Male	70	66	67
Age: years (median)	53–79 (67)	51–79 (67)	53–79 (67)
Smoking: pack-year (median)	27–132 (45)	26–133 (43)	24–138 (48)
**Italian Cohort (SMAC)**			
*n* =	31	31	31
Clinical stage:	-	-	
- IA			10
- IB			4
- IIA			1
- IIB			8
- IIIA			5
- IIIB			1
- IVA			1
- IVB			1
Histopathology:	-	-	
- Adenocarcinoma			24
- Squamous cell carcinoma			3
- Not otherwise specified NSCLC			3
- SCLC			1
- Other cancer (incl. mixed types)			0
Sex:			
- Female	9	13	12
- Male	22	18	19
Age: years (median)	55–78 (62)	51–85 (67)	55–85 (71)
Smoking: pack-year (median)	9–80 (42)	25–100 (45)	10–82 (46)

**Table 2:** Indices of the classification model (mean values and 95% confidence intervals; CI).

Set	Overall Accuracy % (95% CI)	Overall AUC (95% CI)	Specificity % (95% CI)			Sensitivity % (95% CI)		
			Ctr	LN	LC	Ctr	LN	LC
Training	48 (46–50)	0.59 (0.58–0.60)	68 (65–71)	65 (61–68)	61 (58–65)	41 (36–46)	49 (45–54)	53 (51–56)
Test	37 (31–43)	0.60 (0.56–0.65)	57 (48–67)	50 (37–63)	54 (48–60)	29 (19–39)	40 (33–47)	42 (26–59)
Validation	29 (26–31)	0.51 (0.47–0.55)	51 (41–60)	47 (43–53)	38 (35–41)	31 (25–38)	25 (20–30)	29 (23–36)

## Data Availability

The data presented in this study are available on request from the corresponding author. The data are not publicly available due to privacy and ethical reasons.

## Supplementary Materials

The following are available online at https://www.mdpi.com/article/10.3390/cancers13112714/s1, Table S1: Serum metabolites used in quantitative analyses aimed to detect differences between groups of participants of the lung cancer screening program, Table S2: Serum metabolites used in binary analyses (present vs absent) aimed to detect differences among groups, Table S3: Comparison of differences in levels of serum metabolites differentiating groups of participants of the lung cancer screening between the two independent studies, Table S4: Indices of the 3-state classifier based on the signature of specific serum metabolites.
